# Complications of Post-Roux-en-Y Gastric Bypass: A Case of Excluded Stomach Perforation

**DOI:** 10.7759/cureus.75514

**Published:** 2024-12-11

**Authors:** C. Ryan Williams, Nathaniel Grabill, Mena Louis, Deepak Dev Vivekanandan, Timothy Stevens

**Affiliations:** 1 General Surgery, Northeast Georgia Medical Center Gainesville, Gainesville, USA; 2 Trauma and Acute Care Surgery, Northeast Georgia Medical Center Gainesville, Gainesville, USA

**Keywords:** bariatric surgery, gastric bypass, morbid obesity, stomach rupture, ulcer

## Abstract

Roux-en-Y gastric bypass (RYGB) is a common surgical treatment for morbid obesity, but rare complications involving the excluded gastric remnant can pose significant challenges. A 65-year-old female with a history of RYGB presented with sudden onset of left upper quadrant abdominal pain, bloating, nausea, and loss of appetite. Laboratory tests revealed leukocytosis. An initial CT scan showed significant distention of the excluded stomach, suggesting possible obstruction. While preparing for percutaneous decompression, her abdominal pain worsened acutely. A repeat CT scan demonstrated decompression of the excluded stomach and new free fluid in the abdomen, indicating a perforation. Emergent exploratory laparotomy uncovered a large necrotic perforation in the excluded gastric remnant and extensive adhesions from prior surgeries. A partial gastrectomy and antrectomy were performed to remove the perforated tissue. Pathological examination revealed ulcerated gastric mucosa with acute and chronic inflammation, reactive gastropathy, and no evidence of *Helicobacter pylori* infection or malignancy. Postoperatively, the patient recovered well with supportive care and was discharged home. Diagnosing complications in the excluded stomach after RYGB is challenging due to altered anatomy and nonspecific symptoms. Maintaining a high index of suspicion is essential when evaluating post-RYGB patients with unexplained abdominal pain. Early recognition and prompt surgical intervention are critical for favorable outcomes in these patients.

## Introduction

Roux-en-Y gastric bypass (RYGB) is a widely performed surgical procedure for the treatment of morbid obesity and its related comorbidities [1]. This operation involves creating a small gastric pouch from the proximal stomach, gastrojejunostomy, and jejunojejunostomy, bypassing the majority of the stomach and the duodenum [2]. The alteration in anatomy leads to both restrictive and malabsorptive weight loss mechanisms, resulting in significant and sustained weight reduction for many patients [3].

While RYGB has proven benefits, it also carries the risk of various complications, both in the immediate postoperative period and long term [4]. Among these, complications involving the excluded gastric remnant are particularly rare but can be life-threatening [5]. The remnant stomach, although bypassed by the normal flow of food, continues to secrete gastric juices and is subject to the same pathological processes as the rest of the gastrointestinal tract [6]. Conditions such as ulcers, bleeding, distention, and perforation can occur, often presenting diagnostic challenges due to the altered anatomy [7].

Diagnosing issues in the excluded stomach is complicated by the inaccessibility of this portion of the stomach using standard endoscopic techniques [8]. Symptoms may be vague or nonspecific, and patients might not immediately seek medical attention [9]. Imaging studies, such as CT scans, become essential tools but can be difficult to interpret without a high degree of clinical suspicion [10]. Delayed diagnosis can lead to worsening of the condition and increased risk of severe complications [11].

There is limited data on therapeutic approaches to dilation of the excluded stomach post-Roux-en-Y [12]. One case study described using endoscopic ultrasound (EUS) to create a gastro-gastrostomy from the gastric pouch to the gastric remnant [13]. This was achieved by using a lumen-apposing metal stent deployed during EUS [14]. Another technique is to perform a percutaneous gastrostomy of the remnant stomach. A retrospective study of eight patients evaluated this percutaneous approach and found that seven of the eight patients successfully achieved decompression. Of note in this study, all eight patients underwent intervention within one year of the Roux-en-Y procedure. If a patient has had a perforation and is unstable, then exploratory laparotomy becomes the primary method of treatment [15].

Awareness of the potential for serious complications in the gastric remnant is crucial for healthcare providers managing patients post-RYGB6. A high index of suspicion, combined with appropriate diagnostic strategies, can facilitate early detection and intervention. Educating patients about the importance of reporting new or unusual gastrointestinal symptoms promptly is also an important aspect of long-term care after bariatric surgery [16].

## Case presentation

A 65-year-old female presented to the ED with a one-day history of abdominal pain. The pain was located in the left upper quadrant and epigastric area, described as aching and stretching, with intermittent sharp and severe episodes. She also experienced bloating, nausea, dry heaving, and a loss of appetite. She denied any fever, chills, diarrhea, constipation, or recent contact with sick individuals.

Her medical history was limited with the only significant history being hypertension. Although she was morbidly obese prior to the RYGB, she did not have diabetes and associated comorbidities such as gastroparesis. Surgical history included an open RYGB performed in 1998, a hysterectomy in 1991, bilateral salpingo-oophorectomy in 2001, arthroscopic shoulder surgery possibly for rotator cuff repair in 2022, cesarean section in 1986, and breast cyst removal. She did not smoke or consume alcohol.

On review of systems, she reported loss of appetite, bloating, abdominal pain, and nausea. She denied chest pain, shortness of breath, constipation, diarrhea, hematemesis, dysuria, back pain, or neurological symptoms such as headaches, weakness, or numbness.

Physical examination revealed a woman in mild distress due to pain. Vital signs were within normal limits. Abdominal examination showed tenderness in the left upper quadrant and epigastric region without guarding or rebound tenderness. There were no palpable masses, organomegaly, or signs of peritoneal irritation. The rest of the physical examination was unremarkable.

Laboratory studies indicated a white blood cell count of 12.4 K/uL, suggesting leukocytosis. Hemoglobin was 14.1 g/dL, and hematocrit was 41.1%, both within normal limits. The platelet count was 343 K/uL. Electrolytes were normal except for an elevated blood urea nitrogen (BUN) of 42 mg/dL and a glucose level of 151 mg/dL. Liver enzymes and renal function tests were within normal ranges.

**Figure 1:**
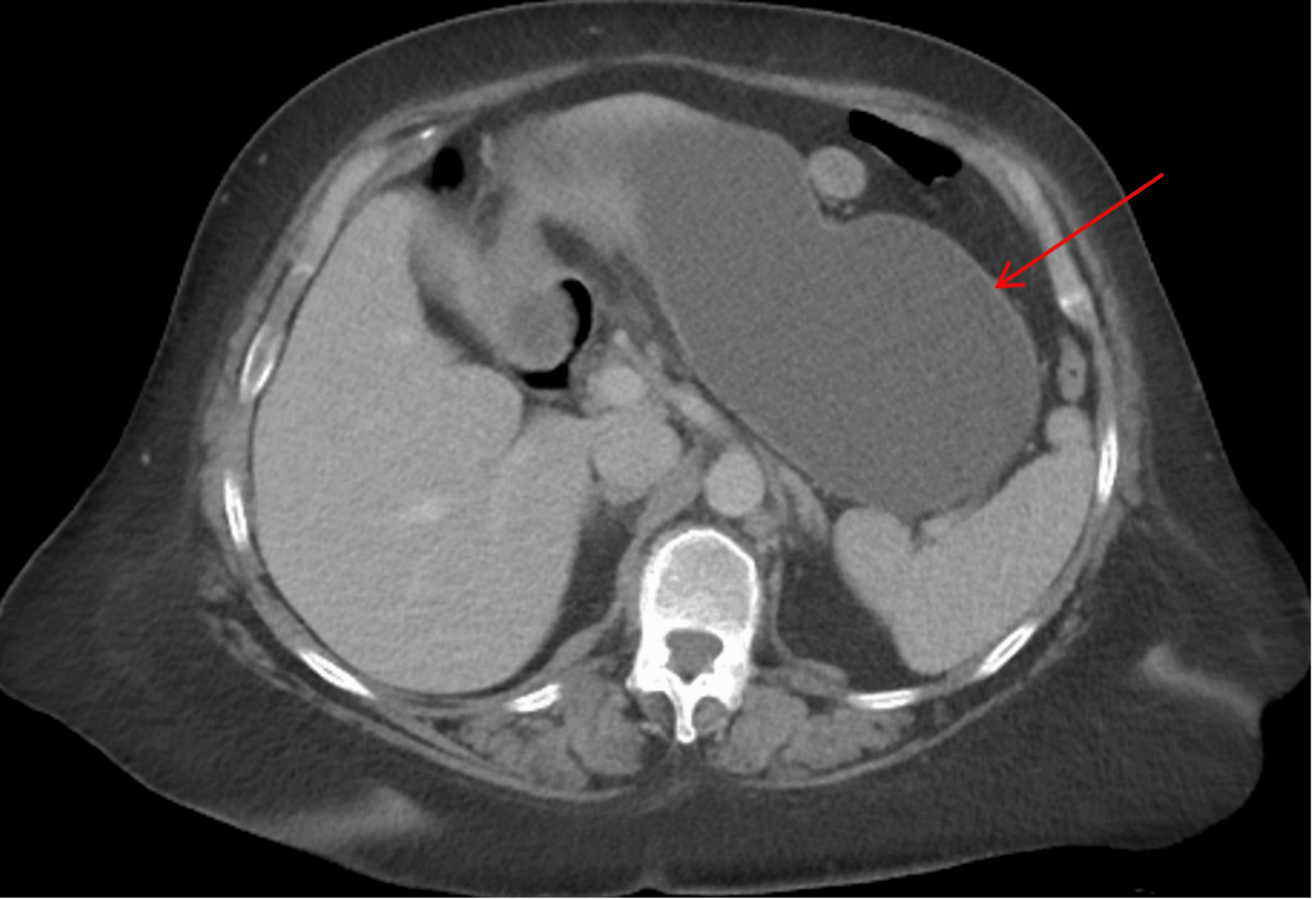
CT abdomen and pelvis with IV contrast (axial view) demonstrates a severely distended excluded stomach (red arrow)

An initial CT scan of the abdomen and pelvis with contrast showed fluid-filled distention of the stomach, particularly the excluded gastric remnant from her previous gastric bypass surgery (Figure 1). No other acute intra-abdominal abnormalities were noted.

**Figure 2:**
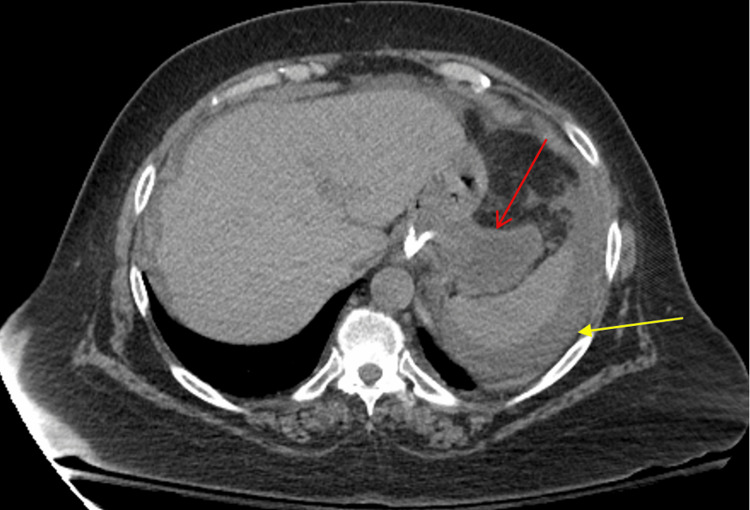
Repeat CT abdomen and pelvis with IV contrast (axial view) performed after an acute change in abdominal pain demonstrates a decompressed gastric remnant (red arrow) with free fluid (yellow arrow) concerning a perforation of the excluded stomach

Given the significant distention of the excluded stomach, plans were made for percutaneous gastrostomy tube placement to decompress it. However, during transportation to the radiology department, the patient experienced a sudden worsening of abdominal pain. A repeat CT scan revealed that the previously distended excluded stomach was now decompressed, with new free fluid present throughout the abdomen and pelvis (Figure 2). These findings raised concern for a perforation of the gastric remnant.

**Figure 3:**
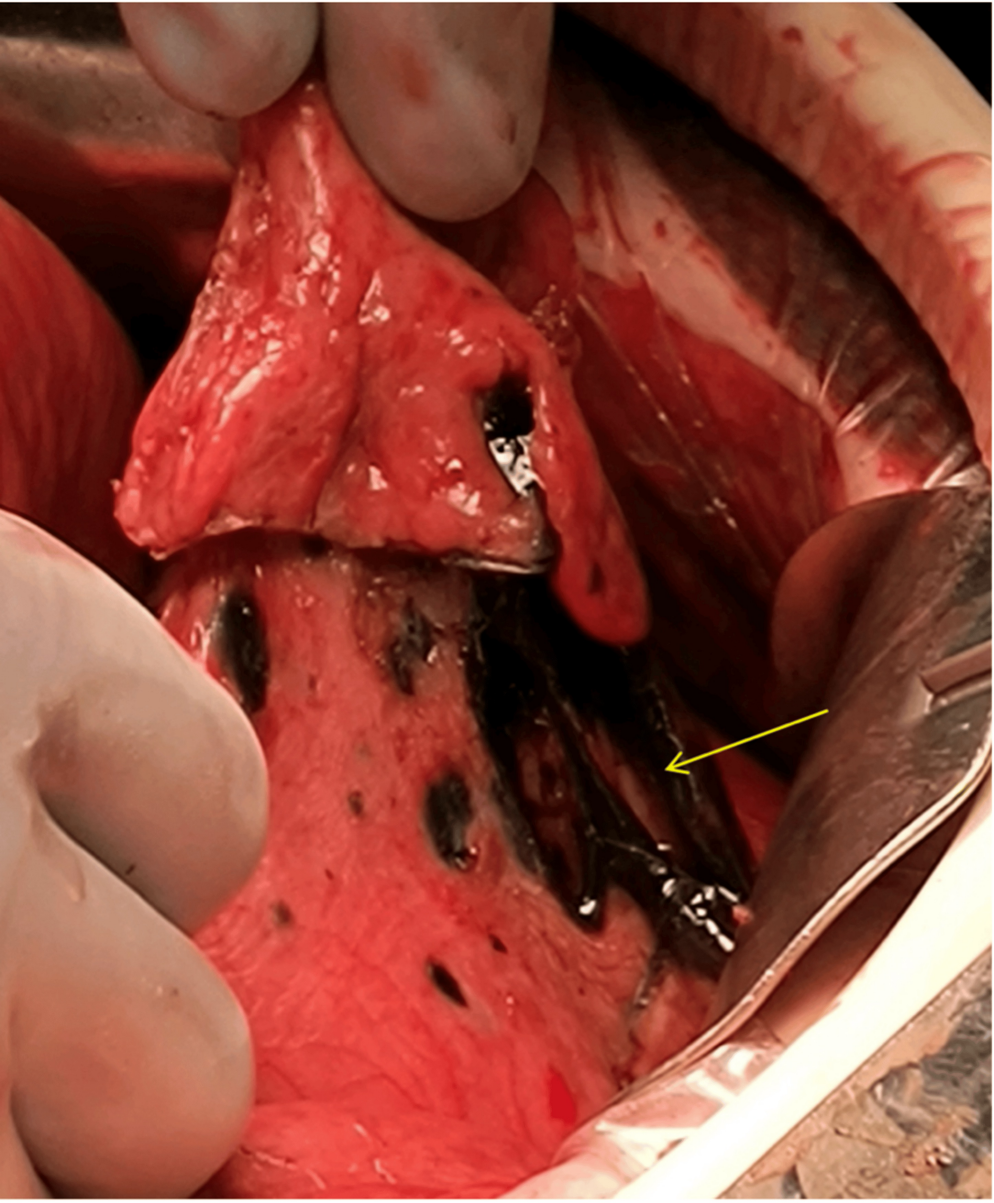
Intraoperative photograph of large necrotic perforation (yellow arrow) in the excluded stomach

An emergent exploratory laparotomy was performed due to the difficulty of handling an expected gastric perforation laparoscopically. Intraoperative findings included extensive adhesions from prior surgeries and a large necrotic perforation in the excluded gastric remnant (Figure 3). Once access to the peritoneum was obtained, the gastric remnant was noted to have a perforated ulcer on the superficial surface of the excluded stomach. To gain adequate exposure for the repair of the defect, the triangular ligament of the liver, transverse colon, and duodenum were mobilized. A palpable mass was also noted in the antrum which was blocking the pylorus. Biopsies were taken from the antrum, and a partial gastrectomy was carried out using a surgical stapler to remove the perforated and necrotic tissue. The specimen was sent for pathological examination. The remainder of the small bowel including the jejunojejunostomy and the large bowel were inspected without further pathology noted.

Pathology reports indicated ulcerated gastric mucosa with acute and chronic inflammation and reactive gastropathy. There was an area of ischemia and full-thickness perforation. Tests for *Helicobacter pylori *were negative, and there was no evidence of intestinal metaplasia, dysplasia, or malignancy.

Postoperatively, the patient was managed in the surgical intensive care unit. She was kept nil per os with IV fluids and antibiotics. Over the following days, her diet gradually advanced from clear liquids to regular food as she tolerated. A Jackson-Pratt drain placed during surgery showed decreasing output and was eventually removed. She remained hemodynamically stable, her pain was well-controlled, and she began ambulating without assistance. She was discharged home with a prescription for a proton pump inhibitor and instructions for close follow-up.

## Discussion

Perforation of the excluded gastric remnant after RYGB is a rare but serious complication that requires prompt recognition and intervention [17]. In the case of the 65-year-old female presented here, subtle symptoms evolved into a life-threatening condition, illustrating the complexities involved in managing long-term complications of bariatric surgery.

RYGB alters the normal gastrointestinal anatomy by creating a small gastric pouch and bypassing the majority of the stomach and duodenum [2]. While this procedure is effective for weight loss and improving comorbid conditions, it leaves the excluded stomach in place [6]. Gastric secretions continue from the remnant stomach but now it is excluded from enteral feeding, making it susceptible to pathological processes that may go unnoticed due to the lack of typical symptoms [7].

In this patient, the initial presentation included left upper quadrant abdominal pain, bloating, nausea, and loss of appetite - all symptoms that could be attributed to a variety of benign conditions. However, laboratory findings of leukocytosis and elevated BUN hinted at an underlying inflammatory or infectious process. The initial CT scan revealed distention of the excluded stomach, an unusual finding that suggested an obstruction or dysfunction at the level of the duodenum or pylorus.

Obstruction can lead to the accumulation of gastric secretions in the remnant stomach, increasing intraluminal pressure [18]. This pressure can compromise mucosal blood flow, resulting in ischemia and ulceration [18]. In the absence of prompt decompression, the risk of perforation escalates [19]. The patient’s sudden worsening of abdominal pain and the appearance of free fluid on repeat CT imaging indicated that a perforation had likely occurred.

Surgical exploration confirmed a large necrotic perforation in the excluded gastric remnant and extensive adhesions from previous surgeries. Adhesions can complicate surgical procedures by obscuring anatomical landmarks and increasing operative time and risk [20]. The perforation was managed by performing a partial gastrectomy and antrectomy to remove the necrotic tissue and control contamination.

Pathological examination showed ulcerated gastric mucosa with acute and chronic inflammation and reactive gastropathy. The absence of *H. pylori* infection and no history of NSAIDs complicated the etiology of the ulcer. The hypothesis was that chronic antral inflammation near the pylorus caused a gastric outlet obstruction, increasing luminal pressure, and causing gastric wall ischemia. Ischemic ulcers can develop when compromised blood flow meets increased pressure within the gastric wall, leading to tissue necrosis.

Postoperative management focused on supportive care, including IV fluids, antibiotics, gradual advancement of diet, and proton pump inhibitor therapy to reduce gastric acid secretion. The patient’s recovery was uneventful, and she was discharged home with instructions for close follow-up.

Education plays a vital role in the long-term care of bariatric patients [16]. They should be informed about potential late complications and encouraged to seek medical attention for new or unexplained symptoms [21]. Regular follow-up appointments provide opportunities to monitor for nutritional deficiencies, assess for gastrointestinal issues, and reinforce lifestyle modifications [22]. Avoiding NSAIDs and other ulcerogenic substances can reduce the risk of ulcer formation in the remnant stomach [23].

Interdisciplinary collaboration among surgeons, radiologists, gastroenterologists, and pathologists is crucial for optimal patient outcomes. In this case, the combined efforts of these specialists facilitated timely diagnosis and effective management of a life-threatening condition. Awareness and proactive management of such rare complications can significantly improve the quality of care for patients who have undergone RYGB11.

## Conclusions

Complications involving the excluded gastric remnant can occur many years after RYGB and may present with nonspecific abdominal symptoms, making timely diagnosis challenging. The altered gastrointestinal anatomy requires clinicians to maintain a high index of suspicion and utilize appropriate imaging modalities to identify such rare but serious conditions. Early recognition and prompt surgical intervention are essential to prevent severe outcomes and improve patient prognosis in cases of remnant gastric perforation.
